# Lactoferrin Gene Expression and Antioxidant Activity in Tomato

**DOI:** 10.3390/biology15141171

**Published:** 2026-07-16

**Authors:** Mingfang Feng, Xinyi Tang, Jinzhu Fan, Aoxue Wang

**Affiliations:** 1 College of Life Sciences, Northeast Agricultural University, Harbin 150030, China; fengmingfang@neau.edu.cn (M.F.); 15289494018@163.com (X.T.); 17548215078@163.com (J.F.); 2 College of Horticulture, Northeast Agricultural University, Harbin 150030, China

**Keywords:** tomato, bioreactor, *hLF*, HFL-1 cells, antioxidation

## Abstract

Plant bioreactors offer low production costs, high biosafety, and facile scalability, making tomato an excellent platform for the expression of bioactive proteins. Lactoferrin (LF) possesses a wide range of biological activities; however, its production using microbial expression systems is limited by inherent drawbacks. These limitations can potentially be overcome through tomato-based heterologous expression. Nevertheless, studies investigating the biological activity of LF produced in tomato expression systems remain scarce, and validation using animal cell models has been relatively limited. In this study, the human lactoferrin (*hLF*) gene was codon-optimized, and the recombinant plasmid pSlhLF was constructed and introduced into tomato plants via *Agrobacterium*-mediated transformation to generate positive transgenic lines. Molecular analyses confirmed that *hLF* was expressed in all examined tomato tissues, with the highest expression level detected in fruits. Most transgenic lines exhibited significantly increased transcript levels of genes encoding antioxidant enzymes. Furthermore, in vitro cell assays demonstrated that tomato-derived hLF promoted the proliferation of H_2_O_2_-treated human fetal lung fibroblasts (HFL-1 cells) and reduced intracellular reactive oxygen species (ROS) accumulation. These findings suggest that hLF expressed in tomato may exert its antioxidant activity, at least in part, through the direct scavenging of ROS.

## Simple Summary

This study used tomatoes to produce human lactoferrin and explored its antioxidant effects. Transgenic tomato lines carrying the target gene exhibited upregulated transcription of antioxidant enzyme genes. The protein extracted from these tomatoes could protect and repair cells damaged by oxidation. The results proved this protein works mainly by removing harmful substances directly. This work has created improved tomato varieties and also offers new ideas for developing healthy foods and functional products using ordinary fruits and vegetables.

## 1. Introduction

Plant bioreactors are production platforms that utilize plant cells, tissues, organs, or whole plants to synthesize bioactive substances, including vaccines, antibodies, therapeutic proteins, and enzymes. These products have broad applications in medicine, industry, and agriculture and possess significant therapeutic and commercial value [1]. Tomato (*Solanum lycopersicum*) is one of the most widely cultivated vegetable crops worldwide. It is characterized by a relatively small genome (950 Mb), a short life cycle (45–100 days), and self-pollination, making it an ideal model species for the generation of transgenic plants and the production of plant-derived biopharmaceuticals. Furthermore, as an edible fresh fruit, tomato can preserve heat-sensitive bioactive compounds from degradation, further enhancing its suitability as a plant bioreactor.

LF is a 78–80 kDa non-heme iron-binding glycoprotein belonging to the transferrin family because of its structural similarity to serum transferrin and its high affinity for Fe^3+^. It is composed of approximately 700 amino acid residues organized into two homologous globular lobes (N- and C-lobes) connected by an *α*-helix, with each lobe consisting of four *β*-sheet-rich subdomains (Figure S1) [2]. Each lobe binds one Fe^3+^ ion, and LF can also chelate other metal ions, including Cu^2+^, Zn^2+^, and Mn^2+^. Depending on its iron saturation status, LF exists either as iron-free *apo*-LF or iron-saturated *holo*-LF, with the latter exhibiting greater resistance to proteolytic degradation [3].

LF was first isolated and purified from bovine milk. It is synthesized and secreted primarily by mammalian mucosal epithelial cells and neutrophils and is widely distributed in tears, saliva, vaginal secretions, semen, nasal and bronchial secretions, bile, and gastric juice. Human and bovine colostrum, as well as mature milk, are the major natural sources of LF. Human lactoferrin (hLF) consists of 711 amino acid residues, whereas bovine lactoferrin (bLF) contains 689 residues. LF orthologs from different mammalian species exhibit a high degree of sequence conservation.

LF possesses a wide range of biological activities, including promoting intestinal iron absorption [4], exerting broad-spectrum antibacterial and antiviral effects [5], mediating anticancer activities [6], regulating immune responses, scavenging reactive oxygen species (ROS) as part of its antioxidant function [7,8], and stimulating the proliferation of intestinal epithelial cells in vitro [9]. Owing to these biological properties, LF has been widely applied as a natural preservative in meat products and as a functional ingredient in infant formula, yogurt, and animal feed.

The diverse biological activities of LF endow it with broad application potential, making the development of LF into bioactive agents with appropriate formulations a long-standing research focus [10]. In recent years, there has been a growing number of studies on LF expression in higher plants. For the convenience of clinical translation, rhLF has been expressed in various expression systems and cell lines, including: (1) Animal models (e.g., *Mus musculus*, *Bos taurus*, *Capra aegagrus hircus*, *Ovis aries*); (2) Plant systems (e.g., *Nicotiana tabacum*, *Solanum lycopersicum*, *Oryza sativa*, *Solanum tuberosum*, *Panax ginseng*, *Pyrus pyrifolia*, *Hordeum vulgare*, *Arabidopsis thaliana*, *Medicago sativa*, *Triticum aestivum*) [11]; (3) Fungal systems (e.g., *Aspergillus niger*, *Pichia pastoris*); and (4) Mammalian and insect cell lines (e.g., BHK cells, Sf9 insect cells, CHO cells).

LF is a multifunctional glycoprotein with unique immunomodulatory properties. Previous studies have suggested that the amount of LF obtained through the daily consumption of dairy products is insufficient to achieve optimal health benefits [12], highlighting the need to develop alternative and sustainable sources of LF. Heterologous expression of hLF in tomato offers several advantages. On the one hand, it provides a promising strategy for developing functional foods with antioxidant, antiviral, anticancer, and immunomodulatory properties. On the other hand, given the roles of LF in enhancing disease resistance and facilitating iron absorption, introducing the *hLF* gene into tomato may increase iron accumulation in transgenic plants, improve disease resistance [13,14], and expand the genetic resources available for tomato breeding.

Therefore, in this study, we established a tomato-based bioreactor system for the heterologous production of hLF. We demonstrated that transgenic tomato lines expressing hLF exhibited enhanced stress tolerance accompanied by increased transcription of antioxidant enzyme-related genes. More importantly, this study is the first to evaluate the biological activity of tomato-derived hLF using mammalian cell models. Cell-based assays further demonstrated that tomato-derived hLF exerts its antioxidant effects primarily through the direct scavenging of ROS rather than by altering total antioxidant capacity (T-AOC) or intracellular H_2_O_2_ levels. Collectively, these findings provide both a theoretical basis and technical support for the development of functional tomato cultivars and the production of recombinant pharmaceutical proteins using plant bioreactor systems, thereby expanding the potential applications of plant-based molecular farming.

## 2. Materials and Methods

### 2.1. Plant Materials and Growth Conditions

The tomato cultivar Ailsa Craig (AC) and *Arabidopsis thaliana* used in this study were maintained and propagated in our laboratory. Tomato plants were grown in pots filled with a mixture of peat and vermiculite (3:1) in an artificial climate chamber under the following conditions: 12 h light cycle, with day/night temperatures maintained at 25 °C/20 °C. Samples were collected at two developmental stages: 4-week-old seedlings were used for partial tissue analyses, whereas plants grown to maturity (45–100 days after transplantation) were used for harvesting roots, stems, leaves, and ripe fruits. *Arabidopsis thaliana* seeds were surface-sterilized in 20% sodium hypochlorite solution supplemented with 0.1% Tween-20 for 15 min, followed by thorough rinsing with sterile deionized water at least five times. Surface-sterilized seeds were sown on half-strength Murashige and Skoog (1/2 MS) medium containing 2% (*w*/*v*) sucrose and solidified with 0.8% (*w*/*v*) agar (pH 5.8). The sown seeds were stratified in the dark at 4 °C for 3 days to break seed dormancy and synchronize uniform germination. Subsequently, the Petri dishes were transferred to a growth chamber and cultured at 22 °C ± 2 °C under a 16 h photoperiod for approximately 7 d. Following germination, seedlings were either used for protoplast isolation and subcellular localization analysis or transplanted to soil for further growth.

### 2.2. Construction of the Plant Expression Vector

The *hLF* gene sequence (GenBank accession No. KT006756.1) was fused to a C-terminal 6× His tag at the C-terminus, resulting in a total length of 2154 bp. The gene was codon-optimized for expression in tomato using an online tool (https://www.novopro.cn/tools/codon-optimization.html, accessed on 8 December 2024) and synthesized by Harbin Boshi Biotechnology Co., Ltd. (Harbin, China). The synthesized gene was cloned into the pBWA(V)HS vector and was designated pSlhLF (i.e., pBWA(V)HS-*hLF*-his) (Method S1). Ten microliters of sequence-verified pSlhLF plasmid was added to 100 μL of competent *Agrobacterium tumefaciens* GV3101 cells, followed by freeze–thaw transformation in a 37 °C water bath. The cells were then added to liquid YEB medium and cultured at 28 °C with shaking at 200 rpm for 5 h. After centrifugation, the supernatant was discarded, and the bacterial pellet was resuspended and spread on solid YEB medium for incubation in the dark at 28 °C for 48–96 h.

### 2.3. Agrobacterium-Mediated Tomato Genetic Transformation

Fully mature seeds were extracted from ripe Ailsa Craig tomato fruits propagated in our laboratory. Fruit pulp was thoroughly washed away with running sterile water, and clean seeds were air-dried at room temperature before use. Tomato seeds were rinsed with sterile water for 2 min, disinfected with 75% ethanol for 40 s, sterilized with 10% sodium hypochlorite solution for 7 min, rinsed with sterile water three times, and soaked in sterile water for 1 h. The disinfected seeds were sown on germination medium, initially cultured in the dark for 3–4 d, and then transferred to a light incubator for 4–5 d post-germination. For germinated tomato seedlings with fully expanded cotyledons, the petioles and cotyledon tips were excised with a scalpel, and the middle parts were cut into 2–3 segments, which were then inoculated on pre-culture medium with the abaxial (lower) surface contacting the medium and incubated at 23 ± 2 °C for 2–3 d. Single colonies of *Agrobacterium* were picked and resuspended in infection buffer to a final OD600 of 0.1. The tomato explants were immersed in the bacterial suspension for 10–15 min, blotted dry with sterile filter paper, and placed on co-culture medium for dark incubation at 23 ± 2 °C for 2 d. After co-cultivation, the explants were transferred to selection medium, where visible callus formed within 7–10 days, followed by continuous selection culture for an additional 21–28 days. The obtained calli were subsequently transferred to differentiation medium and cultured at 23 °C under a 16 h light/8 h dark photoperiod for 30–40 d. When regenerated shoots reached 2–3 cm in height, they were excised from the calli and inoculated on rooting medium, followed by culture under the identical light and temperature conditions for 10–15 d. The formulas for tissue culture media used in genetic transformation are shown in Table S1.

### 2.4. Generation and Selection of Transgenic Lines

A total of 26 independent T0 transgenic tomato plants were generated via Agrobacterium-mediated transformation. Genomic DNA was extracted from 100 mg of young leaf tissues of all 26 putative transgenic lines and wild-type Ailsa Craig (AC) control plants using the CTAB method, followed by PCR-based molecular validation (Method S2). Among them, 12 independent transgenic lines were randomly selected for further characterization via RT-qPCR analysis. Based on transgene expression levels and phenotypic consistency, three representative lines (OE1, OE4, and OE12) were selected for subsequent experiments. T3-generation transgenic plants were used for all analyses.

### 2.5. Quantitative Real-Time Reverse Transcription PCR (RT-qPCR) Detection

To analyze the expression pattern of the *hLF* gene in different organs of transgenic tomatoes, 12 PCR-positive candidate transgenic tomato lines were selected for expression analysis. Tissue samples including roots, stems, young leaves and fully ripe red fruits were harvested at their respective specific developmental stages: roots, stems and leaves were collected at the vegetative stage with 4–5 true leaves, while fruits were sampled at the full red ripening stage. Wild-type tomato tissues collected at identical growth stages were set as parallel control samples. For the transcriptional analysis of antioxidant enzyme-encoding genes in transgenic lines OE1, OE4 and OE12, leaf samples were obtained at the five-leaf one-heart stage, with wild-type tomatoes at the same growth stage serving as controls. Approximately 100 mg of each collected tissue was rinsed with sterile distilled water, blotted dry with filter paper, immediately frozen in liquid nitrogen, and ground into fine powder for subsequent total RNA extraction. Total RNA was extracted using the CTAB-based method, followed by DNase I digestion to eliminate residual genomic DNA contamination (Method S3). Reverse transcription was performed with the iScriptTM gDNA Clear cDNA Synthesis Kit (Bio-Rad Laboratories, Inc., Hercules, CA, USA) in accordance with the manufacturer’s protocol. Quantitative real-time PCR (qPCR) was conducted using the SYBR Premix Ex Taq Kit (Takara Bio Inc., Kusatsu, Shiga, Japan) in a 20 μL reaction mixture, with the following composition: 10 μL of 2× qPCR Mix, 0.5 μL of forward primer (10 μmol·L^−1^), 0.5 μL of reverse primer (10 μmol·L^−1^), 0.5 μL of cDNA template, and 8.5 μL of ddH_2_O. The PCR cycling conditions were as follows: initial pre-denaturation at 95 °C for 3 min; followed by 40 cycles of 95 °C for 10 s, 58 °C for 30 s, and 72 °C for 20 s. A melting curve analysis from 65 °C to 95 °C was conducted after amplification to verify the specificity of amplification of each target fragment. Each sample was analyzed with three biological replicates and three technical replicates. The UBI gene was used as the internal reference gene. Cycle threshold (Ct) values were acquired using an QuantStudio™ 5 Real-Time PCR System (Applied Biosystems, Foster City, CA, USA), and the relative gene expression levels were calculated using the 2^−△△CT^ method. All primer sequences used for RT-qPCR are listed in Table S2.

### 2.6. Western Blot Analysis

The target protein was detected using the following antibodies: His-tag antibody (mouse monoclonal, Proteintech Group, Inc., Rosemont, IL, USA) diluted at a ratio of 1:5000, with goat anti-mouse IgG (ABclonal Technology Co., Ltd., Wuhan, China) as the secondary antibody diluted at a ratio of 1:10,000; hLF antibody (rabbit monoclonal, Abcam plc, Cambridge, UK) used at 1:5000, with goat anti-rabbit IgG (Proteintech Group, Inc., Rosemont, IL, USA) as the secondary antibody diluted at a ratio of 1:10,000. Actin antibody (mouse monoclonal, ABclonal Technology Co., Ltd., Wuhan, China) was used as the loading control, used at 1:5000, with goat anti-mouse IgG (ABclonal Technology Co., Ltd., Wuhan, China) as the secondary antibody diluted at a ratio of 1:10,000. Total proteins were extracted from the roots, stems, leaves, and fruits of different transgenic tomato lines. Resolving gels and stacking gels were prepared, and samples were loaded into the wells in the preset order. The gel was placed in transfer buffer, and PVDF membranes and transfer filter papers of the same size as the separating gel were cut. The PVDF membrane was activated in methanol for 30 s, rinsed with transfer buffer, and equilibrated in transfer buffer; the transfer filter papers were also equilibrated in transfer buffer prior to electrotransfer. After transfer, the membrane was washed in TBS for 5 min and blocked in blocking buffer at 25 °C for 1 h. The membrane was incubated with the diluted primary antibody at 4 °C overnight, followed by washing with TBST three times (10 min each). Then, the membrane was incubated with the diluted secondary antibody at 25 °C for 1 h, washed with TBST three times (10 min each), and temporarily stored in TBS. Equal volumes of ECL luminescent solution A and B were mixed and stored in the dark for later use. The membrane was placed on an exposure platform with the protein side facing up, and the mixed luminescent solution was evenly spread over the entire membrane. Chemiluminescent signals were detected sequentially, and the resulting images were captured and saved for subsequent densitometric analysis.

### 2.7. ELISA Quantitative Detection

Transgenic tomato fruits were homogenized, and ELISA was performed according to the manufacturer’s instructions using a Human Lactoferrin ELISA Kit (Abcam plc, Cambridge, UK). Fifty microliters (μL) of each sample or standard was added to the corresponding wells, followed by 50 μL of antibody mixture. The plate was sealed and incubated on a shaker at 200 rpm for 1 h at room temperature. Each well was washed 3–5 times with 350 μL of 1× washing buffer: the washing buffer was aspirated completely or decanted, and 350 μL of 1× washing buffer was added to each well, with an incubation time of at least 10 s per wash. After the final wash, the plate was inverted and blotted dry with sterile absorbent paper towels. A 100 μL volume of TMB developing solution was added to each well, and the plate was incubated on a shaker at 200rpm for 8 min. One hundred microliters of stop solution was added to each well, and the plate was shaken for 1 min to mix thoroughly. The absorbance (OD) value at 450 nm was measured using a microplate reader.

### 2.8. Subcellular Localization Assay

The constructed plasmid pAN580-*hLF*-linker was transformed into *Arabidopsis thaliana* protoplasts. *Arabidopsis thaliana* seedlings were grown at approximately 25 °C for 25–30 d. Several seedlings were collected and immersed in 5–10 mL of enzymatic hydrolysis solution (sufficient to cover the tissues). Enzymatic hydrolysis was performed at 24 °C for 4 h without shaking. The mixture was filtered through a 40 μm filter, and the filtrate was centrifuged at 10.06 *g* for 3 min to discard the supernatant. The pellet was washed twice with 10 mL of pre-cooled W5 solution, centrifuged at 10.06 *g* for 3 min (centrifugation temperature: 4–25 °C), and resuspended in 500 μL of MMG solution (100 μL of protoplasts per sample). Prior to transformation, protoplast viability and integrity were assessed under a microscope using a 40× objective lens, with 20–40 protoplasts per field of view (to assess protoplast viability and integrity). One hundred microliters of protoplast suspension was mixed with 20 μL of DNA, followed by the addition of 120 μL of PEG4000 solution (equal to the total volume of DNA and protoplasts). The mixture was gently mixed and incubated at room temperature for 30 min. The reaction was terminated by adding 1 mL of W5 solution. Protoplasts were collected by centrifugation at 10.06 *g* for 3 min, and the supernatant was discarded. The pellet was washed 1–2 times with 1 mL of W5 solution, resuspended in 1 mL of W5 solution, and cultured in the dark at 28 °C for 18–24 h. After carefully removing the supernatant while retaining approximately 100 μL of protoplast suspension, the samples were observed using a laser scanning confocal microscope (LSCM, Zeiss, Oberkochen, Germany).

### 2.9. Proliferation Assay of Human Fetal Lung Fibroblast (HFL-1) Cells

Human fetal lung fibroblasts (HFL-1) were purchased from Jiangsu KeyGEN BioTECH Co., Ltd., Nanjing, China. Cell line authentication confirmed the absence of multi-allelic loci. STR profiling confirmed the identity of the HFL-1 cell line. The DSMZ database shows the cell name as HFL1, corresponding to the cell catalog number CCL-153. Screening for multi-allelic alleles and cell line matching was performed as described in Method S4. The full DNA STR profile of this cell line is presented in Figure S2. HFL-1 cells were digested, counted, and resuspended to a concentration of 5 × 10^4^ cells·mL^−1^. One hundred microliters of the cell suspension was added to each well of a 96-well cell culture plate, which was then incubated at 37 °C in a 5% CO_2_ incubator for 24 h. Five experimental groups were set for the H_2_O_2_-induced oxidative stress model:

Blank control group:

Cells cultured in complete medium without H_2_O_2_ or protein treatment.

H_2_O_2_ model group:

Cells treated with 80 μmol·L^−1^ H_2_O_2_ only.

WT tomato protein group:

H_2_O_2_-treated cells supplemented with total protein extracts from wild-type tomato fruits.

Transgenic *hLF* tomato protein group:

H_2_O_2_-treated cells supplemented with total protein extracts from *hLF*-expressing transgenic tomato fruits.

Commercial hLF group:

H_2_O_2_-treated cells supplemented with purified human lactoferrin (Merck KGaA, Darmstadt, Germany).

H_2_O_2_ was diluted to the desired concentration with complete medium, and 100 μL of H_2_O_2_-containing medium was added to each well; a negative control group (treated with an equal volume of complete medium without H_2_O_2_) was set up in parallel. The plate was incubated at 37 °C in a 5% CO_2_ incubator for 24 h. After medium replacement, different proteins were added to the wells, including total proteins from wild-type (WT) tomato fruits, total proteins from *hLF*-transgenic tomato fruits, and standard human lactoferrin (Merck KGaA, Darmstadt, Germany). The proteins were dissolved in 500 μL of medium, filter-sterilized, and directly added to the cells. Prior to cell treatment, the absolute hLF concentration in transgenic tomato total protein extracts was quantified using the human lactoferrin ELISA standard curve (Figure S3). The loading volume of transgenic tomato protein solution was calculated and adjusted to reach a uniform final hLF concentration of 1 ng·mL^−1^ in all culture systems, consistent with the dosage of commercial pure hLF. An equal volume of total protein extract from WT tomato fruits was supplemented into the WT treatment group to minimize potential interference from endogenous tomato proteins among all groups. The plate was incubated at 37 °C in a 5% CO_2_ incubator for 24 h. CCK-8 staining was performed: the medium was discarded, and 100 μL of medium containing 10% CCK-8 solution (Beyotime Biotechnology, Shanghai, China) was added to each well. The plate was incubated for another 2 h in the incubator, shaken gently for 10 min to mix, and the OD value was measured at 450 nm using a microplate reader. The cell proliferation rate was calculated for each group based on the OD values.

### 2.10. Reactive Oxygen Species (ROS) Detection Assay

DCFH-DA was diluted with serum-free medium at a ratio of 1:1000 to a final concentration of 10 μM. After removing the spent cell culture medium, an appropriate volume of diluted DCFH-DA working solution was added to completely cover the cells. The cells were incubated at 37 °C in a cell culture incubator for 20 min, then washed three times with serum-free medium to remove excess extracellular DCFH-DA. Intracellular ROS levels were detected using a microplate reader with an excitation wavelength of 495 nm and an emission wavelength of 529 nm.

### 2.11. Total Antioxidant Capacity (T-AOC) Assay

The FRAP working solution was pre-incubated at 37 °C and used within 1–2 h. For cell samples, approximately 1 × 10^6^ cells were collected and resuspended in 200 μL of ice-cold PBS (pH 7.4). The cells were homogenized to fully disrupt cell membranes and release intracellular antioxidant components, followed by centrifugation at 12,000× *g* at 4 °C for 5 min. The supernatant was collected for subsequent analyses. A 27.8 mg quantity of FeSO_4_·7H_2_O, provided with the kit, was dissolved in an appropriate solvent and adjusted to a final volume of 1 mL to prepare a 100 mmol·L^−1^ stock solution. The stock solution was serially diluted to concentrations of 0.15, 0.3, 0.6, 0.9, 1.2, and 1.5 mmol·L^−1^ (diluted with distilled water or sample preparation buffer). One hundred and eighty microliters (180 μL) of FRAP working solution was added to each well of a 96-well plate. Five microliters (5 μL) of distilled water or PBS was added to blank control wells; a 5 μL volume of FeSO_4_ standard solutions (at the aforementioned concentrations) was added to standard curve wells; a 5 μL volume of each sample or Trolox solution (0.15–1.5 mmol·L^−1^, as the positive control) was added to sample wells. The plate was gently shaken and incubated at 37 °C for 3–5 min, and the absorbance (OD) value was measured at 593 nm using a microplate reader. The T-AOC of samples was calculated based on the standard curve and expressed as FeSO_4_ standard solution equivalent concentrations.

### 2.12. Hydrogen Peroxide (H_2_O_2_) Detection Assay

For cultured cells, cells were collected into centrifuge tubes, and the supernatant was discarded. Lysis buffer was added at a ratio of 100–200 μL per 1 × 10^6^ cells, followed by thorough homogenization to lyse the cells and release intracellular contents. The mixture was centrifuged at 12,000× *g* at 4 °C for 3–5 min, and the supernatant was collected for subsequent assays. Because H_2_O_2_ is unstable, its actual concentration was calibrated prior to the assay. A series of H_2_O_2_ standard solutions was prepared at concentrations ranging from approximately 1 to 100 μmol·L^−1^, and the absorbance (OD) value was measured at 240 nm using a 96-well UV–visible microplate reader. The standard solutions were diluted with H_2_O_2_ detection lysis buffer to concentrations of 0, 1, 2, 5, 10, 20, 50, and 100 μmol·L^−1^ to generate a standard curve. The H_2_O_2_ detection reagent was thawed on ice prior to use. Fifty microliters of sample or standard was added to each detection well, followed by 100 μL of H_2_O_2_ detection reagent. The plate was gently mixed, incubated at room temperature (15–30 °C) for 30 min, and the OD value was measured immediately at 560 nm. The H_2_O_2_ concentration in samples was calculated based on the standard curve.

### 2.13. Statistical Analysis

All experiments were performed with three independent biological replicates unless otherwise stated. Data are presented as the mean ± standard error (SE). Statistical analyses were performed using GraphPad Prism version 9. Comparisons between two groups were conducted using Student’s *t*-test, whereas differences among multiple groups were analyzed by one-way analysis of variance (ANOVA) followed by Tukey’s post hoc test. Differences were considered statistically significant at *p* < 0.05.

For experiments involving the HFL-1 oxidative stress model, including the control, H_2_O_2_-treated, WT tomato protein-treated, transgenic *hLF* tomato protein-treated, and commercial hLF-treated groups, one-way ANOVA followed by Tukey’s multiple-comparison test was applied. Cell proliferation, ROS accumulation, total antioxidant capacity (T-AOC), and intracellular H_2_O_2_ levels were analyzed using the same statistical procedure.

## 3. Results

### 3.1. Transformation of Tomato with the hLF Gene

**Figure 1:**
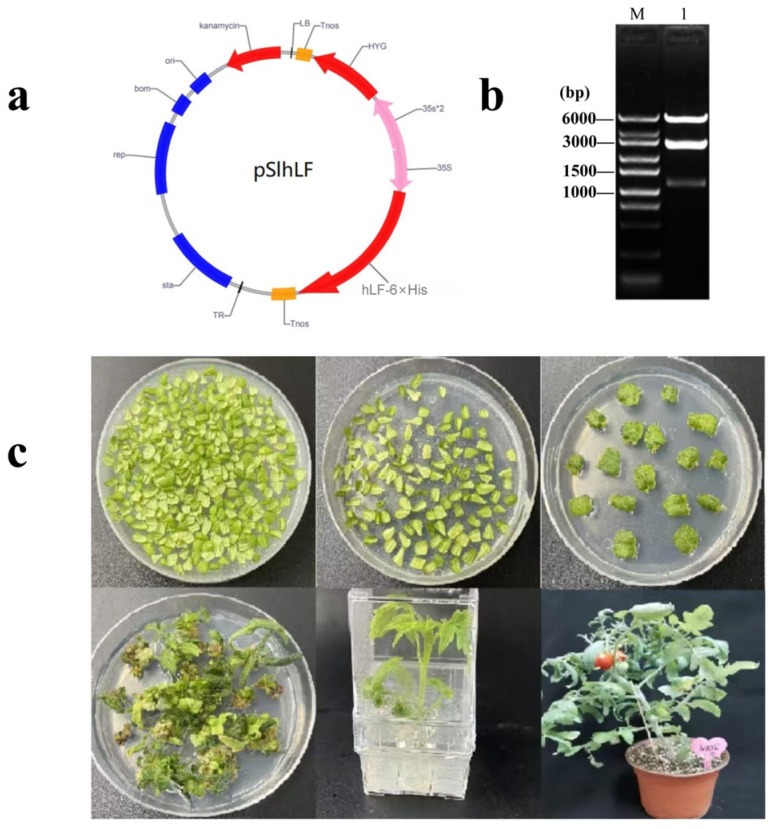
Construction of *hLF*-transgenic tomato plants. (**a**) Schematic diagram of recombinant plasmid pSlhLF. (**b**) Restriction enzyme digestion analysis of recombinant plasmid pSlhLF using EcoRV. M: DL 6000 DNA Marker; 1: EcoRV-digested pSlhLF. (**c**) Key steps of *Agrobacterium*-mediated tomato transformation: the sequential transformation process from (**top-left**) to (**bottom-right**): cotyledon pre-culture, co-cultivation, resistant screening, adventitious bud differentiation, root induction, and soil acclimatization.

A recombinant expression plasmid, designated pSlhLF, was successfully constructed (Figure 1a). The recombinant plasmid was subsequently verified by single-enzyme digestion with EcoRV (Figure 1b). A sequencing fragment of approximately 3000 bp was analyzed, and the sequencing results showed 100% identity with the designed sequence. The recombinant plasmid was then introduced into tomato plants via *Agrobacterium*-mediated transformation to generate transgenic lines carrying the *hLF* gene (Figure 1c).

**Figure 2:**
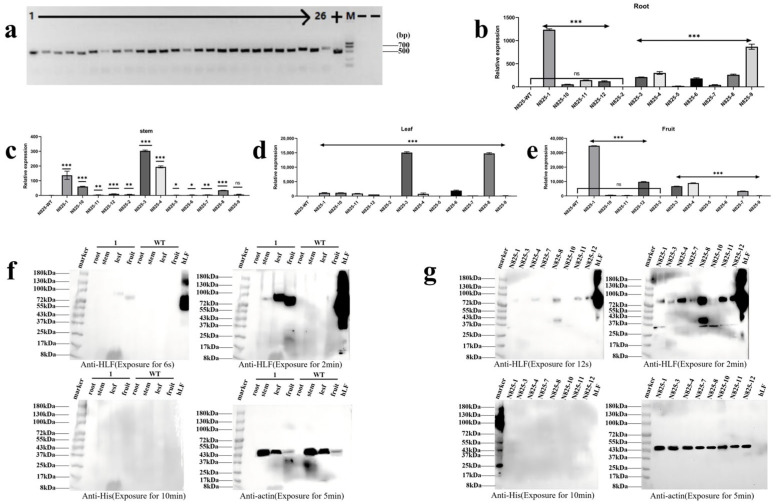
Molecular and protein-level identification of *hLF*-transgenic tomato plants. (**a**) PCR detection of *hLF*-transgenic tomato plants. WT: wild type (replaced with “−” in the figure); P: plasmid DNA control (replaced with “+” in the figure); M: DL 1000 DNA marker; 1–26: 26 independent *hLF*-transgenic tomato lines. (**b**–**e**) RT-qPCR analysis of *hLF* transcript levels in *hLF*-transgenic tomato plants. 1–12: 12 independent *hLF*-transgenic tomato lines; WT: wild-type tomato plants. No-template control (NTC) reactions were used as zero blanks to eliminate background signals from non-specific amplification. The gene expression level of wild-type (WT) samples was normalized to 1 as the reference control. Relative gene expression was quantified using the 2^−ΔΔCT^ method. All data are presented as the mean ± standard error of three independent biological replicates. * *p* < 0.05, ** *p* < 0.01, *** *p* < 0.001. ns, no significant difference. Two-tailed Student’s *t*-test with unequal variance was performed for pairwise comparisons using GraphPad Prism 9. (**f**) Immunoblot detection of hLF protein accumulation in roots, stems, leaves and fruits of transgenic tomato line No. 1 overexpressing hLF (1 represents transgenic tomato line 1 overexpressing hLF; WT: wild type). (**g**) Immunoblot detection of hLF protein accumulation in fruits from different independent hLF-overexpressing transgenic tomato lines.

Genomic DNA was extracted from 26 putative transgenic tomato plants using the CTAB method, followed by PCR analysis to identify positive transformants (Figure 2a). The expression pattern of *hLF* in different organs of the transgenic tomato plants was subsequently analyzed by RT-qPCR. The results showed that *hLF* was expressed in roots, stems, leaves, and fruits, with the highest transcript level detected in fruits (Figure 2b–e; Table S3).

Roots, stems, leaves, and fruits from transgenic line OE1 were collected for Western blot analysis (Figure 2f). No immunoreactive band was detected using the anti-His antibody. In contrast, distinct bands were detected in stems, leaves, and fruits using the anti-hLF antibody, with the strongest signal observed in fruits. Western blot analysis of fruits from different transgenic lines further confirmed the presence of hLF protein in all tested lines (Figure 2g).

In the present study, recombinant hLF protein was detected using an anti-hLF antibody, and the observed immunoreactive band corresponded to the expected molecular weight of the full-length protein (approximately 80 kDa). However, no signal was detected with the anti-His antibody. Several factors may account for the absence of the anti-His signal, including steric hindrance, epitope masking caused by protein folding, or post-translational modifications that reduce the accessibility of the C-terminal His tag.

To quantify hLF accumulation in transgenic tomato fruits, a standard curve was generated using serial dilutions of purified hLF purchased from Merck KGaA (Darmstadt, Germany) (Figure S3). ELISA analysis demonstrated that all examined transgenic lines expressed hLF protein, although substantial differences in protein accumulation were observed among the individual lines (Table S4).

In addition, subcellular localization analysis showed that hLF was localized to the endoplasmic reticulum, nucleus, and cytoplasm (Figure S4).

### 3.2. Physiological Analysis of hLF-Transgenic Tomatoes

Total RNA was extracted from the leaves of wild-type (WT) tomato plants and three independent hLF-transgenic lines (OE1, OE4, and OE12). RT-qPCR was performed to quantify the transcript levels of five antioxidant enzyme genes, including catalase (CAT), glutathione S-transferase (GST), glutathione peroxidase (GPX), glutathione reductase (GR), and superoxide dismutase (SOD) (Table S5).

**Figure 3:**
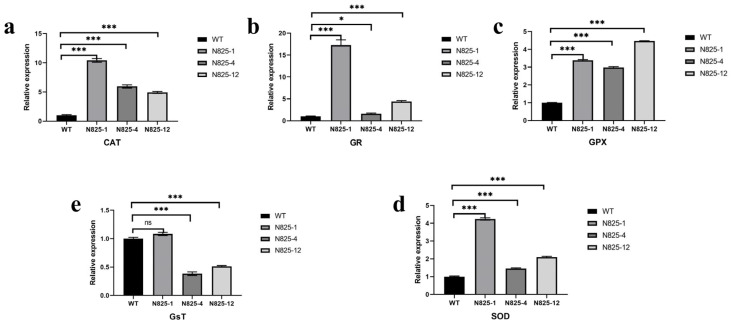
Transcript levels of antioxidant enzyme-encoding genes in *hLF*-transgenic tomato plants. (**a**–**e**) Relative expression levels of key antioxidant enzyme-encoding genes in wild-type (WT) and hLF-transgenic tomato lines. (**a**) Relative expression of CAT; (**b**) Relative expression of GR; (**c**) Relative expression of GPX; (**d**) Relative expression of SOD; (**e**) Relative expression of GsT. No-template control (NTC) reactions were used as zero blanks to eliminate background signals from non-specific amplification. The gene expression level of wild-type (WT) samples was normalized to 1 as the reference control. Relative gene expression was quantified using the 2^−ΔΔCT^ method. All data are presented as the mean ± standard error of three independent biological replicates. Two-tailed Student’s *t*-test with unequal variance was performed for pairwise comparisons using GraphPad Prism 9. * represents *p* ≤ 0.05, *** represents *p* ≤ 0.001, and ns represents *p* > 0.05.

Compared with the WT control, GST transcript levels were lower in transgenic lines OE4 and OE12. In contrast, the transcript levels of all other antioxidant enzyme genes were significantly higher in the transgenic lines than in the WT plants (Figure 3).

### 3.3. Activity of the hLF Produced in Transgenic Tomatoes

HFL-1 cells were first exposed to a series of H_2_O_2_ concentrations to establish an oxidative stress model, and the optimal H_2_O_2_ concentration was determined based on cell viability (Table S6). Treatment with 80 μmol·L^−1^ H_2_O_2_ induced an appropriate level of oxidative injury and was therefore selected for subsequent assays evaluating the biological activity of hLF produced in transgenic tomatoes.

The inhibition rates of HFL-1 cells in the WT tomato, *hLF*-transgenic tomato, and commercial hLF groups were all lower than that observed in the 80 μmol·L^−1^ H_2_O_2_ treatment group (23.15%). ANOVA was performed to compare the WT tomato, *hLF*-transgenic tomato, commercial hLF, and H_2_O_2_ treatment groups. No significant difference was observed between the WT tomato group and the H_2_O_2_ treatment group (*p* > 0.05), whereas both the *hLF*-transgenic tomato group and the commercial hLF group showed significantly lower inhibition rates than the H_2_O_2_ treatment group (*p* < 0.05). These results indicate that hLF produced in transgenic tomatoes effectively promotes the proliferation of HFL-1 cells subjected to oxidative stress (Table 1).

Intracellular ROS, T-AOC, and H_2_O_2_ levels were subsequently determined using the established oxidative stress model (80 μmol·L^−1^ H_2_O_2_). Standard curves for T-AOC and H_2_O_2_ quantification are presented in Figures S5 and S6.

ROS levels in the WT tomato, *hLF*-transgenic tomato, and commercial hLF groups were all lower than those in the H_2_O_2_ treatment group (1175.90 relative fluorescence units). One-way ANOVA revealed that only the commercial hLF group differed significantly from the H_2_O_2_ treatment group (*p* < 0.05), whereas no significant differences were detected in either the WT tomato or *hLF*-transgenic tomato groups (*p* > 0.05). Furthermore, no significant difference was observed between the commercial hLF and *hLF*-transgenic tomato groups (*p* > 0.05) (Table 2 and Table S7). These findings indicate that tomato-derived hLF exhibits ROS-scavenging activity comparable to that of commercial hLF.

Compared with the H_2_O_2_ treatment group (T-AOC = 1.11), no significant differences in T-AOC were observed among any of the treatment groups (*p* > 0.05), although the commercial hLF group showed a numerically higher T-AOC value than the H_2_O_2_ treatment group (Table 3).

**Figure 4:**
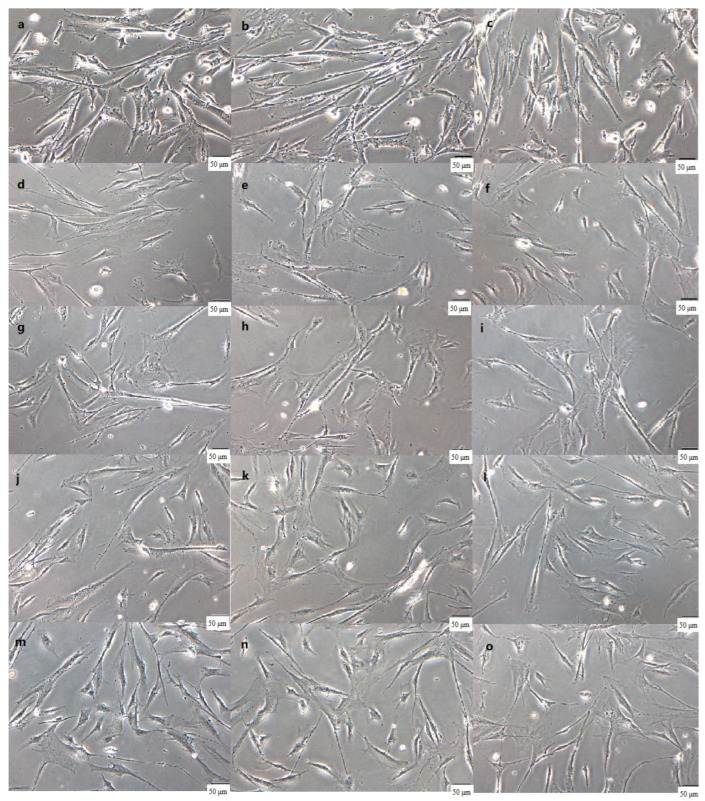
T-AOC Determination and Observation of HFL-1 Cell Morphology. (**a**–**c**): Control group, cells cultured with complete medium only, no H_2_O_2_ or protein treatment; (**d**–**f**): 80 μmol·L^−1^ H_2_O_2_ treatment group; (**g**–**i**): 80 μmol·L^−1^ H_2_O_2_ + WT tomato treatment group; (**j**–**l**): 80 μmol·L^−1^ H_2_O_2_ + *hLF*-transgenic tomato treatment group; (**m**–**o**): 80 μmol·L^−1^ H_2_O_2_ + standard hLF treatment group. All groups included three biological replicates. Scale bar = 50 μm.

Cell morphology was simultaneously examined during the T-AOC assay. Compared with the untreated control (Figure 4a–c), H_2_O_2_ treatment markedly reduced cell density and induced a slender morphology accompanied by increased cellular branching (Figure 4d–f). Treatment with commercial hLF following H_2_O_2_ exposure restored cell density and improved cell morphology compared with the H_2_O_2_ treatment group (Figure 4m–o). Similarly, treatment with either WT tomato protein extracts (Figure 4g–i) or *hLF*-transgenic tomato protein extracts (Figure 4j–l) improved cell proliferation relative to the H_2_O_2_ treatment group (Figure 4d–f), although the effects were less pronounced than those observed with commercial hLF. Overall, all three treatments alleviated H_2_O_2_-induced cellular injury, with commercial hLF exhibiting the greatest protective effect.

Intracellular H_2_O_2_ levels in both the WT tomato and commercial hLF groups were significantly lower than those in the H_2_O_2_ treatment group (24.09; *p* < 0.05). In contrast, no significant difference was observed between the *hLF*-transgenic tomato group and the H_2_O_2_ treatment group (*p* > 0.05) (Table 4).

## 4. Discussion

### 4.1. Key Antioxidant Enzymes Examined and Their Biological Functions

Following the successful generation of *hLF*-transgenic tomato lines, RT-qPCR was performed to quantify the transcript levels of five antioxidant enzyme genes, namely CAT, GST, GPX, GR, and SOD. These enzymes play distinct but complementary roles in scavenging ROS and maintaining cellular redox homeostasis. CAT decomposes metabolically generated H_2_O_2_ into water and oxygen, thereby alleviating oxidative damage and enhancing stress tolerance [15]. GST catalyzes glutathione conjugation reactions, which are essential for detoxifying reactive compounds and maintaining intracellular redox balance [16,17]. GPX functions as a ubiquitous plant peroxidase involved in antioxidant defense by reducing hydrogen peroxide and lipid hydroperoxides [18]. GR regenerates reduced glutathione through an NADPH-dependent reduction reaction, thereby sustaining the major intracellular antioxidant pool required for ROS detoxification [19]. SOD constitutes the first line of enzymatic antioxidant defense by catalyzing the conversion of superoxide radicals into H_2_O_2_ and O_2_, thereby minimizing oxidative damage caused by reactive oxygen species [20]. Collectively, the established physiological functions of these enzymes provide a framework for interpreting the differential expression patterns observed among the independent *hLF*-transgenic tomato lines.

### 4.2. Association Between Iron Homeostasis and the Expression of Antioxidant Enzyme Genes

Ferrous iron (Fe^2+^) promotes the generation of hydroxyl radicals (·OH) through the Fenton reaction, thereby inducing oxidative stress. Conversely, iron also serves as an essential cofactor for several antioxidant enzymes, including CAT, APX, and Fe-SOD [21]. Therefore, iron homeostasis and antioxidant defense are tightly coordinated in plants and other organisms.

In *Escherichia coli*, iron transport genes and SOD genes are regulated by common transcriptional regulators [22]. In yeast, *MAC1* regulates the expression of ferric reductase and cytoplasmic CAT [23]. In *Brassica napus*, excess iron induces APX expression [24], whereas iron deficiency promotes glutathione biosynthesis but suppresses GST activity in sugar beet roots [25,26]. Similarly, transgenic tobacco expressing *hLF* exhibits reduced transcript levels of GR, GST, APX, and CAT [27]. Together, these previous findings suggest that iron-dependent regulatory networks may underlie the changes in antioxidant enzyme gene expression observed in the present study.

### 4.3. Effects of hLF Transformation on Antioxidant Enzyme Gene Expression in Tomatoes

In the present study, RT-qPCR was used to determine the transcript levels of CAT, GST, GPX, GR, and SOD in *hLF*-transgenic tomato plants. Compared with the WT control, most transgenic lines exhibited increased expression of these antioxidant enzyme genes, whereas reduced GST transcript levels were observed in a small number of lines. This variation may reflect differences in *hLF* expression levels among independent transgenic events [28,29,30].

The reduced GST transcript abundance is consistent with previous observations in *hLF*-transgenic tobacco [27], suggesting that hLF expression may influence glutathione homeostasis. Because iron is required for the activity of several antioxidant enzymes involved in ROS detoxification [31], it is possible that the iron-binding capacity of hLF contributes to the transcriptional regulation of antioxidant enzyme genes in transgenic tomato plants. Furthermore, because all five antioxidant enzymes participate in protecting plants against oxidative stress, their elevated transcript levels in most transgenic lines suggest that hLF expression may enhance the endogenous antioxidant defense system of tomato.

Nevertheless, transcript abundance alone does not necessarily reflect enzymatic activity or stress tolerance. Additional physiological analyses and whole-plant oxidative stress assays will be required to determine whether hLF expression ultimately enhances oxidative stress tolerance in transgenic tomato plants.

### 4.4. Known Mechanisms Underlying the Antioxidant Activity of Lactoferrin (LF)

LF is an iron-binding glycoprotein belonging to the transferrin family and exhibits a wide range of biological activities, including immunomodulatory, anti-apoptotic, antioxidant, iron-chelating, antibacterial, antiviral, and anticancer functions. Recombinant LF has been heterologously expressed in several plant species, including tobacco, tomato, rice, potato, ginseng, pear, barley, *Arabidopsis thaliana*, alfalfa, and wheat [32]. Most previous studies have focused on the establishment of plant expression systems and the antibacterial activity of LF, whereas its antioxidant properties have received relatively little attention [33]. In particular, few studies have evaluated the antioxidant activity of plant-derived hLF using mammalian cell models, a gap addressed by the present study.

Oxidative stress occurs when the production of ROS exceeds the capacity of endogenous antioxidant defense systems, resulting in cellular and tissue damage [34]. Antioxidant defenses consist of both enzymatic and non-enzymatic components, the combined activity of which is commonly evaluated as total antioxidant capacity (T-AOC). Although ROS function as essential signaling molecules under normal physiological conditions, excessive accumulation caused by mitochondrial metabolism or environmental stress can induce oxidative injury. Major ROS include superoxide anions, hydroxyl radicals (·OH), and H_2_O_2_ [35,36,37].

LF exhibits potent antioxidant activity primarily through iron chelation and direct ROS scavenging. Because of its high affinity for iron ions, LF sequesters free iron and inhibits the Fenton reaction, thereby reducing the generation of hydroxyl radicals, one of the most damaging ROS. In addition, LF directly scavenges H_2_O_2_ and suppresses lipid peroxidation [38,39]. Previous studies have further shown that LF can alleviate oxidative damage by upregulating antioxidant-related genes, such as GPx4, and by suppressing the activation of apoptosis-related enzymes [40]. Notably, iron-unsaturated (*apo*-) LF generally exhibits stronger antioxidant activity than iron-saturated (*holo*-) LF [41,42].

A variety of cell-based studies have demonstrated the protective effects of LF against oxidative stress. In gingival fibroblasts, human umbilical vein endothelial cells (HUVECs), and intestinal epithelial cells, LF reduces intracellular ROS accumulation, promotes cell proliferation, and attenuates H_2_O_2_-induced apoptosis [43,44]. In addition, LF protects neurons, mesenchymal stem cells, and osteoblasts by preserving mitochondrial function and delaying cellular senescence. LF-conjugated nanomaterials have also been shown to further enhance its ROS-scavenging and cytoprotective activities [45].

Collectively, the antioxidant mechanisms of LF can be summarized as follows: (1) LF chelates free iron ions, thereby inhibiting the Fenton reaction and reducing hydroxyl radical generation [46,47,48]; (2) LF directly scavenges multiple ROS [49,50,51]; (3) LF suppresses lipid peroxidation through a conserved nine-amino-acid core peptide motif, thereby reducing the accumulation of lipid peroxidation products such as malondialdehyde (MDA); and (4) LF attenuates neutrophil-mediated oxidative burst responses.

### 4.5. Validation of the Antioxidant Activity of Tomato-Derived hLF and Its Proposed Mechanism

H_2_O_2_ is one of the major intracellular sources of ROS and possesses strong oxidative properties. It is generated by multiple oxidase systems, readily diffuses across biological membranes, and oxidizes a wide range of biomolecules. Consequently, H_2_O_2_ is widely used to establish in vitro models of oxidative stress [52,53]. Accordingly, H_2_O_2_ was used in the present study to induce oxidative stress in HFL-1 cells in order to evaluate the antioxidant and cytoprotective activities of tomato-derived hLF.

HFL-1 cells were selected because, as non-transformed primary cells with stable redox homeostasis, they provide a physiologically relevant model for investigating oxidative stress responses in normal human tissues. Moreover, HFL-1 cells have been widely used in studies of lactoferrin bioactivity and avoid the altered antioxidant signaling commonly observed in tumor-derived cell lines [54].

During optimization of the oxidative stress model, the inhibition rate increased with increasing H_2_O_2_ concentration, whereas excessive H_2_O_2_ exposure resulted in irreversible cellular damage. Therefore, 80 μmol·L^−1^ H_2_O_2_ was selected for subsequent experiments [46].

**Figure 5:**
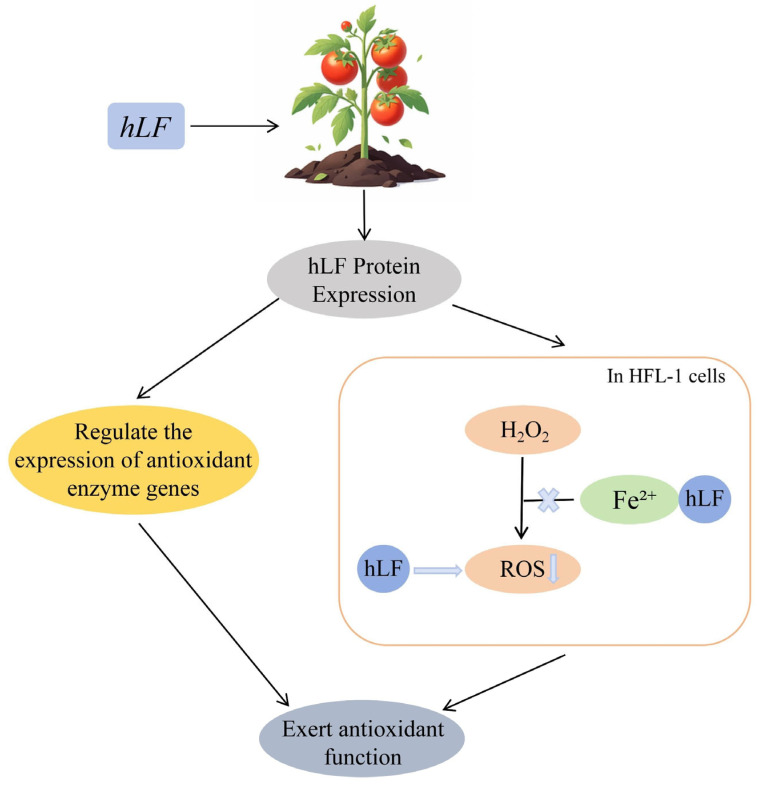
Proposed hypothetical mechanism underlying the antioxidant activity of hLF-transgenic tomato plants.

Our results demonstrated that tomato-derived hLF promoted the proliferation of H_2_O_2_-treated HFL-1 cells and reduced intracellular ROS accumulation. In contrast, no significant effects were observed on either T-AOC or intracellular H_2_O_2_ levels compared with the H_2_O_2_-treated control group. These findings suggest that hLF produced in tomato primarily exerts its antioxidant activity through the direct scavenging of ROS rather than by altering the overall antioxidant capacity of the cells. Figure 5 illustrates the proposed hypothetical mechanism underlying the antioxidant function of hLF-transgenic tomatoes.

### 4.6. Potential Agronomic Applications

The present study demonstrated that most *hLF*-transgenic tomato lines exhibited significantly increased transcript levels of key antioxidant enzyme genes. In addition, in vitro experiments using HFL-1 cells showed that hLF extracted from transgenic tomatoes alleviated H_2_O_2_-induced oxidative injury by promoting cell proliferation and reducing intracellular ROS accumulation, although no significant changes were observed in T-AOC or intracellular H_2_O_2_ levels. Together, these molecular and cellular findings provide preliminary evidence supporting the potential agronomic value of these transgenic materials, despite the absence of direct stress tolerance assays in tomato plants. Nevertheless, this study has one important limitation. All experiments were conducted using a single tomato cultivar, which limits the generalizability of our findings to other tomato genotypes and plant species. Further studies involving multiple tomato cultivars will be necessary to validate the broader applicability of these results.

Under environmental stresses such as drought, salinity, and pathogen infection, excessive ROS rapidly accumulate in plant cells, leading to lipid peroxidation, nucleic acid damage, impaired photosynthesis, and ultimately growth inhibition, leaf wilting, and tissue necrosis [55]. The two complementary molecular responses observed in the present study suggest that *hLF*-transgenic tomatoes may possess enhanced stress tolerance. First, the consistently elevated expression of antioxidant enzyme genes (CAT, SOD, GPX, and GR) may strengthen the endogenous enzymatic antioxidant system and thereby improve the capacity to detoxify ROS under adverse environmental conditions. Second, the HFL-1 cell assays demonstrated that tomato-derived hLF possesses direct ROS-scavenging activity, which may reduce secondary ROS production through iron chelation and inhibition of the Fenton reaction.

Nevertheless, these proposed mechanisms remain hypothetical and should not be interpreted as direct evidence of enhanced stress tolerance in tomato plants. Future studies involving drought, salinity, and pathogen challenge assays will be required to determine whether *hLF* overexpression confers improved stress resistance under physiological and field conditions. Despite this limitation, the present study expands the potential agricultural applications of plant-derived *hLF* and provides a theoretical basis for developing stress-tolerant tomato germplasm through plant-based molecular farming.

## 5. Conclusions

In this study, the *hLF* gene was codon-optimized according to the codon usage preference of tomato, and the recombinant expression vector pSlhLF was successfully constructed. The recombinant vector was introduced into tomato explants via *Agrobacterium*-mediated transformation. Molecular and biochemical analyses, including PCR, RT-qPCR, Western blotting, and ELISA, confirmed the stable integration and expression of the *hLF* gene in transgenic tomato lines. *hLF* transcripts and protein were detected in roots, stems, leaves, and fruits, with the highest expression levels observed in fruits. Significant variation in hLF expression was also detected among independent transgenic lines. Subcellular localization analysis showed that hLF was localized to the ER, nucleus, and cytoplasm. In addition, most transgenic lines exhibited increased transcript levels of antioxidant enzyme genes, including CAT, GST, GPX, GR, and SOD, compared with WT plants. These findings suggest that hLF expression may enhance the endogenous antioxidant system of tomato through transcriptional regulation of antioxidant-related genes, although further whole-plant stress assays are required to determine whether these molecular changes translate into improved oxidative stress tolerance.

In vitro assays further demonstrated that tomato-derived hLF promoted the proliferation of HFL-1 cells subjected to H_2_O_2_-induced oxidative stress while reducing intracellular ROS accumulation. In contrast, no significant effects were observed on T-AOC or intracellular H_2_O_2_ levels. These results suggest that tomato-derived hLF primarily exerts its antioxidant activity through direct ROS scavenging rather than by enhancing the endogenous antioxidant defense system or suppressing intracellular ROS production pathways.

Overall, this study successfully generated transgenic tomato lines expressing biologically active hLF and demonstrated their potential as a plant-based platform for the production of functional recombinant proteins. In addition to modulating the expression of antioxidant enzyme genes, tomato-derived hLF exhibited antioxidant activity in a mammalian cell model, highlighting its potential value for the development of functional tomato products. Furthermore, this work provides a useful experimental framework for the heterologous expression of other pharmaceutical proteins in tomato and expands the potential applications of plant bioreactor systems for the production of high-value bioactive proteins.

## Figures and Tables

**Table 1:** Effects of *hLF*-Transgenic Tomato-Derived hLF on the Inhibition Rate of H_2_O_2_-Stressed HFL-1 Cells.

Group	Control	80 μmol·L^−1^ H_2_O_2_	80 μmol·L^−1^ H_2_O_2_ + WT	80 μmol·L^−1^ H_2_O_2_ + *hLF*-Transgenic Tomato	80 μmol·L^−1^ H_2_O_2_ + Standard hLF (1 ng·mL^−1^)
OD	1.47	1.13	1.13	1.15	1.20
	1.46	1.13	1.13	1.17	1.19
	1.49	1.13	1.14	1.15	1.19
Mean	1.47	1.13	1.14 ^ns^	1.16 *	1.19 *
SD	0.02	0.00	0.01	0.01	0.01
Inhibition rate (%)	0.00	23.15%	22.78%	21.41%	18.93%

Data are expressed as mean ± SD (*n* = 3). One-way ANOVA followed by Tukey’s multiple comparison test. * *p* < 0.05 versus 80 μmol·L^−1^ H_2_O_2_ group; ns, no significant difference (*p* > 0.05).

**Table 2:** ROS Levels in H_2_O_2_-Stressed HFL-1 Cells.

Group	Control	80 μmol·L^−1^ H_2_O_2_	80 μmol·L^−1^ H_2_O_2_ + WT	80 μmol·L^−1^ H_2_O_2_ + *hLF*-Transgenic Tomato	80 μmol·L^−1^ H_2_O_2_ + Standard hLF (1 ng·mL^−1^)
Fluorescence intensity	31,342	44,315	42,508	40,993	41,226
	30,443	42,181	41,223	42,808	41,056
	31,573	42,392	42,820	41,628	40,541
Mean	31,119.33	42,962.67	42,183.67 ^ns^	41,809.67 ^ns^	40,941.00 *
SD	597.00	1175.90	846.46	921.04	356.69

Data are expressed as mean ± SD (*n* = 3). One-way ANOVA followed by Tukey’s multiple comparison test. * *p* < 0.05 versus the 80 μmol·L^−1^ H_2_O_2_ single treatment group; ns, no significant difference (*p* > 0.05).

**Table 3:** T-AOC Levels in H_2_O_2_-Stressed HFL-1 Cells.

Group	OD	Protein Concentration (mg·mL^−1^)	T-AOC (mmol·g^−1^)	T-AOC Mean Value (mmol·g^−1^)
HFL-1-control-1	0.28	0.52	1.90	1.78
HFL-1-control-2	0.29	0.70	1.73	
HFL-1-control-3	0.29	0.63	1.71	
HFL-1 + H_2_O_2_-1	0.28	0.47	1.06	1.11
HFL-1 + H_2_O_2_-2	0.28	0.50	1.16	
HFL-1 + H_2_O_2_-3	0.27	0.33	1.10	
HFL-1 + H_2_O_2_ + WT-1	0.28	0.44	1.08	1.09 ^ns^
HFL-1 + H_2_O_2_ + WT-2	0.29	0.59	1.03	
HFL-1 + H_2_O_2_ + WT-3	0.27	0.40	1.18	
HFL-1 + H_2_O_2_ + *hLF*-transgenic tomato-1	0.28	0.45	1.00	1.10 ^ns^
HFL-1 + H_2_O_2_ + *hLF*-transgenic tomato-2	0.30	0.72	1.05	
HFL-1 + H_2_O_2_ + *hLF*-transgenic tomato-3	0.27	0.41	1.25	
HFL-1 + H_2_O_2_ + Standard hLF-1	0.29	0.62	1.27	1.21 ^ns^
HFL-1 + H_2_O_2_ + Standard hLF-2	0.28	0.49	1.19	
HFL-1 + H_2_O_2_ + Standard hLF-3	0.27	0.40	1.18	

Data represent mean T-AOC value (*n* = 3 independent replicates). One-way ANOVA followed by Tukey’s multiple comparison test. ns, no significant difference vs. H_2_O_2_-only group (*p* > 0.05).

**Table 4:** Intracellular H_2_O_2_ Concentrations in H_2_O_2_-Stressed HFL-1 Cells.

Group	OD	H_2_O_2_ Concentration (μmol·L^−1^)	H_2_O_2_ Concentration Mean (μmol·L^−1^)
HFL-1-control-1	0.19	12.24	13.20
HFL-1-control-2	0.20	14.13	
HFL-1-control-3	0.19	13.22	
HFL-1 + H_2_O_2_-1	0.27	25.63	24.09
HFL-1 + H_2_O_2_-2	0.26	24.10	
HFL-1 + H_2_O_2_-3	0.25	22.54	
HFL-1 + H_2_O_2_ + WT-1	0.28	26.49	23.79 *
HFL-1 + H_2_O_2_ + WT-2	0.26	23.78	
HFL-1 + H_2_O_2_ + WT-3	0.24	21.11	
HFL-1 + H_2_O_2_ + *hLF*-transgenic tomato-1	0.26	24.67	24.67 ^ns^
HFL-1 + H_2_O_2_ + *hLF*-transgenic tomato-2	0.26	23.24	
HFL-1 + H_2_O_2_ + *hLF*-transgenic tomato-3	0.27	26.10	
HFL-1 + H_2_O_2_ + Standard hLF-1	0.24	20.24	20.87 *
HFL-1 + H_2_O_2_ + Standard hLF-2	0.25	22.02	
HFL-1 + H_2_O_2_ + Standard hLF-3	0.24	20.35	

Data represent raw OD values, individual intracellular H_2_O_2_ concentrations and averaged mean concentrations (*n* = 3 independent biological replicates). One-way ANOVA followed by Tukey’s multiple comparison test was used for statistical analysis. * *p* < 0.05 versus the single 80 μmol·L^−1^ H_2_O_2_ treatment group; ns, no significant difference (*p* > 0.05).

## Data Availability

All data are included in the manuscript.

## Supplementary Materials

The following supporting information can be downloaded at: https://www.mdpi.com/article/10.3390/biology15141171/s1, Table S1: Tissue culture medium for genetic transformation of tomato; Table S2: RT-qPCR detection primers; Table S3: RT-qPCR: Expression pattern of the *hLF* gene in different organs of transgenic tomatoes; Table S4: hLF Protein Content in *hLF*-Transgenic Tomato Fruits; Table S5: RT-qPCR: Transcript levels of five antioxidant enzyme genes; Table S6: Effects of Different Concentrations of H_2_O_2_ on the Inhibition Rate of HFL-1 Cells; Table S7: ROS levels; Figure S1: Schematic structure of LF; Figure S2: The complete STR DNA profile of the cell line; Figure S3: OD450 values were determined using standard hLF at different concentrations to generate a standard curve; Figure S4: Subcellular Localization Analysis of hLF; Figure S5: Standard Curve; Figure S6: H_2_O_2_ determination standard curve; Method S1: Construction of Recombinant Plasmid pSlhLF (pBWA(V)HS-*hLF*-his); Method S2: Genomic DNA extraction and PCR identification of transgenic tomato plants; Method S3: Total RNA extraction; Method S4: Screening for multi-allelic alleles and cell line database matching.

## Abbreviations

The following abbreviations are used in this manuscript:

LF	Lactoferrin
hLF	human Lactoferrin
bLF	bovine Lactoferrin
ROS	Reactive Oxygen Species
BHK cells	Baby Hamster Kidney cells
SF9 insect cells	Spodoptera Frugiperda 9 cells
CHO cells	Chinese Hamster Ovary cells
AC	Ailsa Craig
RT-qPCR	Reverse Transcription quantitative Real-time Polymerase Chain Reaction
LSCM	Laser Scanning Confocal Microscope
HLF-1	Human Fetal Lung Fibroblast
WT	Wild-type
T-AOC	Total Antioxidant Capacity
CAT	Catalase
GST	Glutathione S-transferase
GPX	Glutathione Peroxidase
GR	Glutathione Reductase
SOD	Superoxide Dismutase
MDA	Malondialdehyde
