# Supplementary material for: Lactoferrin Gene Expression and Antioxidant Activity in Tomato
Source: Biology (Basel). 2026 Jul 16;15(14):1171. doi: 10.3390/biology15141171 (PMC13405588; doi:10.3390/biology15141171)
Supplement: Supplementary file 1 [file biology-15-01171-s001.zip › Supplementary Files.pdf]

Tables

Supplemental Table S1. Tissue culture medium for genetic transformation of tomato

Name	Components (per Liter)
Seed Germination Medium	MS powder 4.43 + Sucrose 30 + Agar 7.0
Explant Pre-culture Medium	MS 4.43 + Sucrose 30 + Agar 7.0 + 6-BA 1.0 mg/L + NAA 0.1 mg/L
Agrobacterium Co-cultivation Medium	MS 4.43 + Sucrose 30 + Agar 7.0 + 6-BA 1.0 mg/L + NAA 0.1 mg/L + Acetosyringone (AS) 100 µM
Resistant Selection Medium	MS 4.43 + Sucrose 30 + Agar 7.0 + 6-BA 1.0 mg/L + NAA 0.1 mg/L + Cefotaxime (Cef) 500 mg/L + Hygromycin B 50 mg/L
Adventitious Bud Differentiation Medium	MS 4.43 + Sucrose 30 + Agar 7.0 + 6-BA 1.5 mg/L + NAA 0.1 mg/L + Cefotaxime 300 mg/L + Hygromycin B 40 mg/L
Root Induction Medium	1/2 MS 2.21 + Sucrose 15 + Agar 7.0 + NAA 0.2 mg/L + Cefotaxime 200 mg/L

Supplemental Table S2. RT-qPCR detection primers

target gene	primer	ID
hLF	F:GCACTCGTTCTCAAGGGTGA<br>R:TCTAACAACAGCAACGGCCA	KT006756.1
CAT	F:CAGCCATGCTACTCAGGACC<br>R:GGCTGCAAAGGCAAGATGTC	NM_001247257.2
GPX	F:AATGGTCTGCACTCGCTTCA<br>R:ACCATCTCCAAAGAACCCACC	NM_001247638.2
GR	F:AATTTTGGGGCTTCGGTTGC<br>R:TACACATCCCCGAAGCACAC	NM_001247314.2
GST	F:GCTCGTTTTGTTGATGGCAAG<br>R:TACCACCGATTCAACTCCCT	NM_001247293.1

SOD	F:GGTGTTAGTGGCACCATCCT<br>R:AGCACCATGCTCCTTACCAG	NM_001311084.1
UBI	F:TCGTAAGGAGTGCCCTAATGCTGA<br>R:CAATCGCCTCCAGCCTTGTTGTAA	NM_001346406.1

Supplemental Table S4. hLF Protein Content in *hLF*-Transgenic Tomato Fruits

NO.	OD_{450}	corrected concentration	C_{hLF}（$pg·mL^{-1}$）
WT	0.17	0.03	0.57
1	0.23	0.09	188.88
3	0.21	0.08	143.55
4	0.23	0.09	209.81
7	0.20	0.06	91.24
8	0.35	0.22	631.77
10	0.19	0.06	73.80
11	0.27	0.14	352.79
12	0.27	0.13	349.30

Supplemental Table S6. Effects of Different Concentrations of H_2O_2 on the Inhibition Rate of HFL-1 Cells

Group	Control	H_2O_2 concentration（$\mu mol·L^{-1}$）			
		80	85	90	95
	1.11	1.13	0.75	0.67	0.58
OD	1.06	1.13	0.73	0.66	0.65
	1.11	1.13	0.73	0.69	0.63
Mean	1.10	1.13^{ns}	0.74*	0.67*	0.62*
SD	0.03	0.00	0.01	0.02	0.04
Inhibition rate（%）	0.00	23.15%	32.79%	38.60%	43.24%

Data are shown as mean ± SD (n=3 independent replicates). One-way ANOVA followed by

Tukey’s multiple comparison test. * $p < 0.05$ versus Control group; ns, no significant difference ($p > 0.05$).

Figures

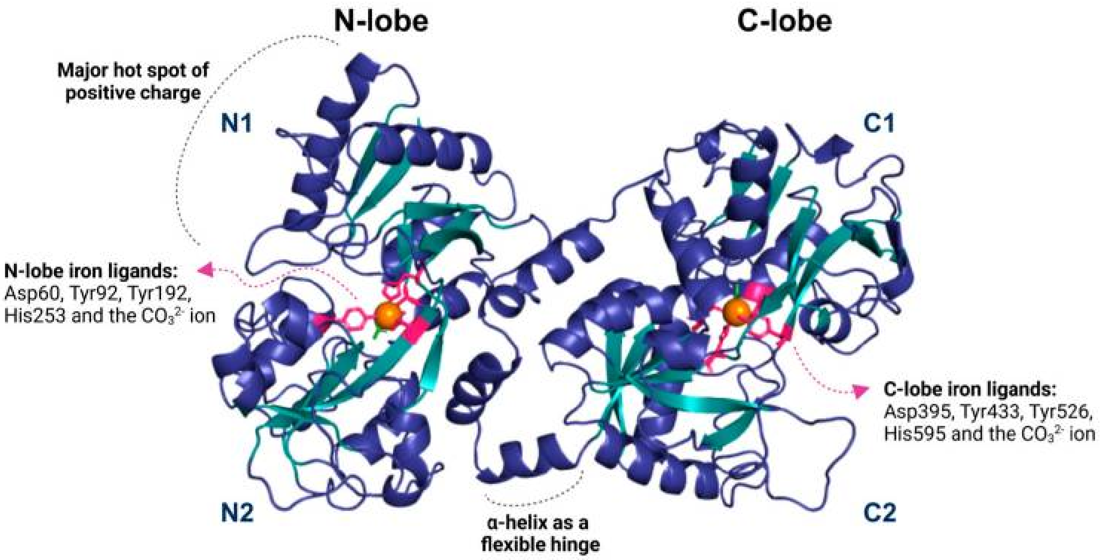

Supplemental Figure S1. Schematic structure of LF.

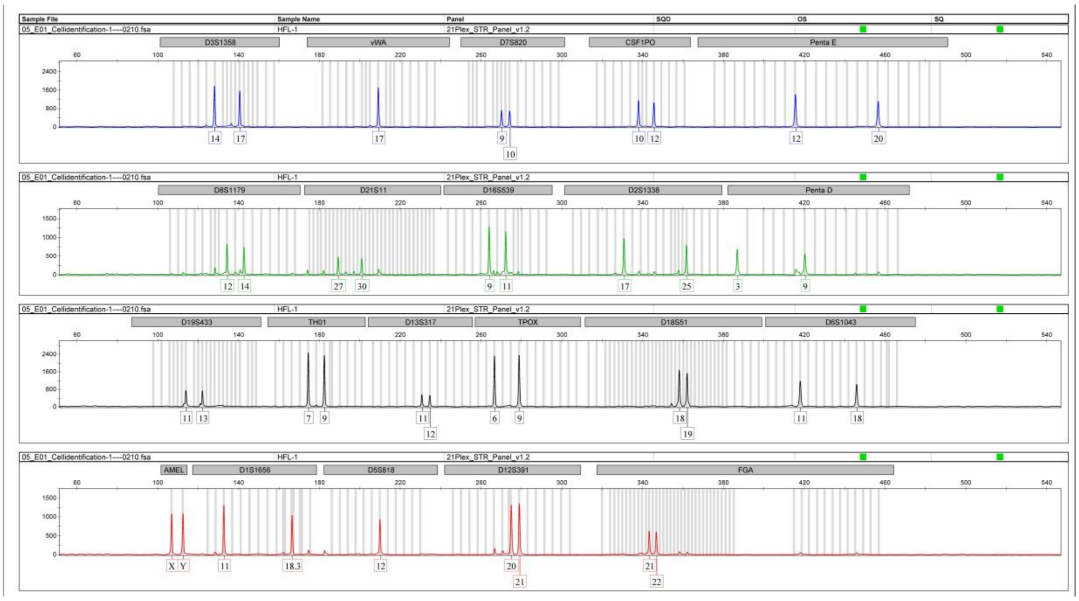

Supplemental Figure S2. The complete STR DNA profile of the cell line

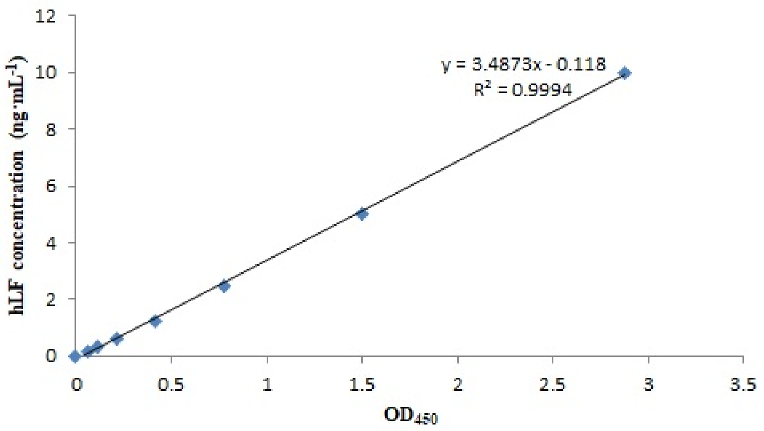

Supplemental Figure S3. OD450 values were determined using standard hLF at different concentrations to generate a standard curve

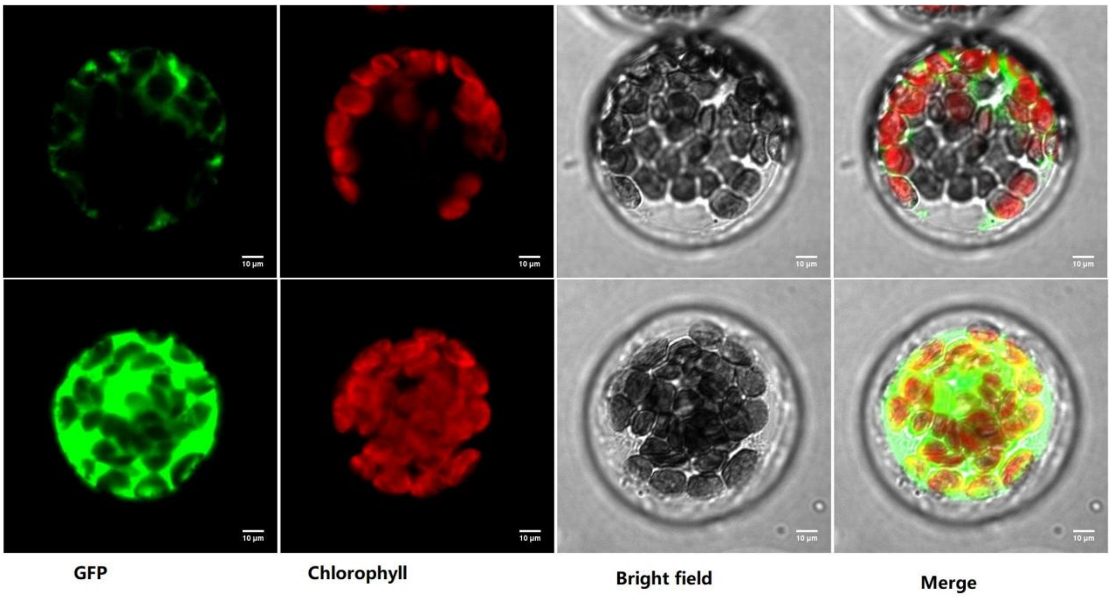

Supplemental Figure S4. Subcellular Localization Analysis of hLF

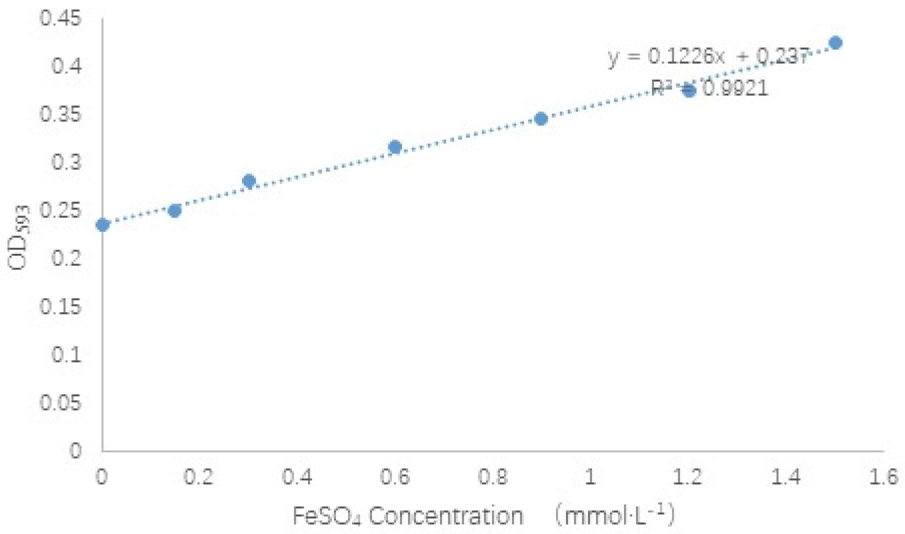

Supplemental Figure S5. Standard Curve

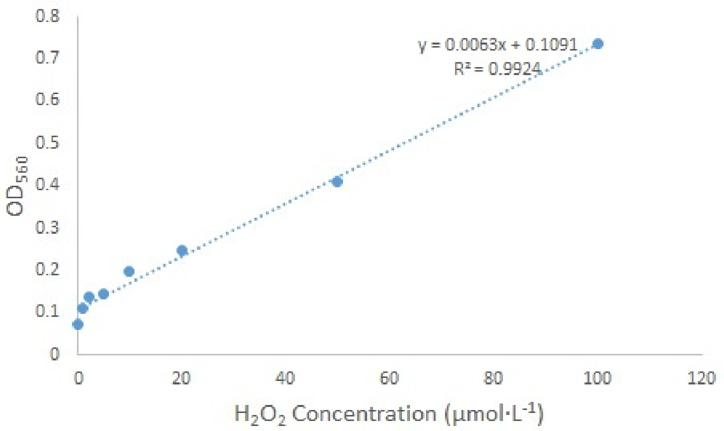

Supplemental Figure S6. H_2O_2 determination standard curve

Methods

Supplemental Method S1. Construction of Recombinant Plasmid pSlhLF (pBWA(V)HS-hLF-his)

1. Primer Synthesis

One pair of specific primers targeting codon-optimized human lactoferrin (hLF) was chemically synthesized:

hLF forward primer (hLF (+)): 5'-atttggagagaacacgggggactttgcaacgccaccatgaagctcgtttttttgg-3'

hLF reverse primer (hLF (-)): 5'-ggcccagtactgaagacagagctagttacattagtgatggtggtggtggtgtttt-3'

2. Amplification of hLF Insert Fragment via PCR

A 50 µL PCR reaction system was prepared to amplify the target hLF gene fragment, and the amplification procedure was set as shown below.

2.1 PCR Reaction System

**Component**	**Volume**
Nuclease-free Water	20 µL
Biorun Pfu PCR Mix	25 µL
hLF (+) primer (100 µM)	2 µL
hLF (-) primer (100 µM)	2 µL
Template DNA (codon-optimized synthetic hLF gene)	1 µL
Total volume	50 µL

2.2 Thermal Cycling Program

1. Initial denaturation: 94 °C, 5 min (1 cycle)

2. Amplification cycle (30 cycles):
Denaturation: 94 °C, 30 sec
Annealing: 50 °C, 45 sec
Extension: 72 °C, 130 sec
3. Final extension: 72 °C, 10 min (1 cycle)
4. Hold: 16 °C, 30 min (1 cycle)
2.3 Gel Electrophoresis and DNA Recovery

The PCR product was separated on a 1% agarose gel at a constant voltage of 5 V/cm for 20 min. The target band corresponding to the 2160 bp hLF fragment was excised under UV light. The DNA fragment was recovered using a commercial gel extraction kit following the manufacturer's standard protocol. The recovered DNA pellet was dissolved in 40 μL nuclease-free water and designated as rDNAG1, which was verified and stored for subsequent recombination with the linearized vector.

3. Linearization of pBWA(V)HS Vector via Restriction Digestion

The backbone vector pBWA(V)HS was digested with BsaI/Eco31I to generate linearized vector fragments.

3.1 Digestion System (20 μL total)

**Component**	**Volume**
Nuclease-free Water	13 μL
10× restriction enzyme buffer	2 μL
BsaI/Eco31I restriction enzyme	1 μL
pBWA(V)HS plasmid DNA	4 μL
Total volume	20 μL

3.2 Digestion Condition

The reaction mixture was incubated at 37 °C for 1 h. After digestion, the linearized vector product was purified with a PCR purification kit and named pBWA(V)HS-ccdB(D) for the subsequent homologous recombination reaction.

4. Homologous Recombination Reaction

The recovered hLF insert rDNAG1 and linearized vector pBWA(V)HS-ccdB(D) were assembled using Biorun 2×EasyClone Mix.

4.1 Recombination System (20 μL total)

**Component**	**Volume**
Nuclease-free Water	0 μL
Biorun 2×EasyClone Mix	10 μL
rDNAG1 (purified hLF insert)	5 μL
Linearized pBWA(V)HS-ccdB(D) vector	5 μL

Component	Volume
Total volume	20 μL

4.2 Recombination Incubation Condition
The reaction system was incubated at 37 °C for 30 h to complete homologous recombination.

5. Transformation of Recombinant Products into E. coli Competent Cells

5–10 μL of the recombination mixture was transformed into E. coli competent cells following standard chemical transformation protocols for E. coli. After transformation, the bacterial suspension was spread onto LB solid plates containing kanamycin resistance, followed by incubation at 37 °C for 12 h to obtain single colonies.

6. Colony PCR Identification of Positive Recombinant Clones

Ten independent single colonies were picked for simultaneous liquid culture and colony PCR validation. The identification primer pair for pBWA(V)HS-hLF was HS35seq/35seq(G) (forward) and NOSseq-R/NOSseq(G) (reverse). Each PCR reaction was prepared in a 25 μL system.

6.1 Colony PCR Reaction System

Component	Volume
Nuclease-free Water	9.5 μL
Biorun Magic PCR Mix	12.5 μL
HS35seq/35seq(G) primer (100 μM)	1 μL
NOSseq-R/NOSseq(G) primer (100 μM)	1 μL
Bacterial colony template	1 μL
Total volume	25 μL

6.2 Thermal Cycling Program
1. Initial denaturation: 94 °C, 5 min (1 cycle)
2. Amplification cycle (30 cycles):
Denaturation: 94 °C, 30 sec
Annealing: 50 °C, 45 sec
Extension: 72 °C, 130 sec
3. Final extension: 72 °C, 10 min (1 cycle)
4. Hold: 16 °C, 30 min (1 cycle)

The target amplified fragment of positive clones was approximately 2260 bp. One to three bacterial cultures corresponding to positive bands were selected: 100 μL of each culture was sent for Sanger sequencing, and the remaining 400 μL was inoculated into 5–10 mL kanamycin-resistant LB liquid medium for shaking culture. After sequencing verification, the clone with fully matched sequence was selected for large-scale plasmid extraction, and the verified recombinant plasmid was designated pSlhLF (pBWA(V)HS-hLF-his) for subsequent tomato transformation experiments.

Supplemental Method S2. Genomic DNA extraction and PCR identification of transgenic tomato plants

Genomic DNA was extracted from young tomato leaf tissues using a modified cetyltrimethylammonium bromide (CTAB) method. Briefly, approximately 100 mg fresh leaf tissue was frozen in liquid nitrogen and fully ground into fine powder with a pre-cooled mortar and pestle. The tissue powder was rapidly transferred into a 2 mL microcentrifuge tube preloaded with 1 mL preheated (65 °C) 2× CTAB extraction buffer (100 mM Tris-HCl pH 8.0, 1.4 M NaCl, 20 mM EDTA, 2% w/v CTAB) supplemented with 20 μL fresh β-mercaptoethanol to inhibit phenolic oxidation in tomato tissues. The mixture was vortexed thoroughly and incubated in a 65 °C water bath for 40 min, with gentle inversion mixing every 10 min during incubation.

After cooling to room temperature, an equal volume of chloroform:isoamyl alcohol (24:1, v/v) was added, followed by gentle inversion for 10 min to form a uniform emulsion. The tube was centrifuged at 12,000 × g for 10 min at room temperature. The clear upper aqueous phase containing DNA was carefully transferred to a new sterile tube without disturbing the intermediate protein layer.

For DNA precipitation, 0.7 volumes of ice-cold isopropanol were added and mixed slowly by inversion, then incubated at −20 °C for 30 min. The mixture was centrifuged at 12,000 × g for 10 min to collect DNA pellets. The supernatant was discarded, and the pellet was washed twice with 70% ice-cold ethanol (each wash 5 min centrifugation at 12,000 × g) to remove residual salts and CTAB impurities.

After complete ethanol removal, the DNA pellet was air-dried at room temperature for 10–15 min (avoid over-drying). The purified genomic DNA was dissolved in 50–80 μL nuclease-free TE buffer (10 mM Tris-HCl, 1 mM EDTA, pH 8.0). RNase A (1 μL, 10 mg/mL) was added to eliminate RNA contamination, followed by incubation at 37 °C for 30 min. DNA concentration and purity were determined using a micro-spectrophotometer, and intact DNA integrity was verified by 1% agarose gel electrophoresis. Qualified genomic DNA was stored at −20 °C for subsequent PCR identification of transgenic tomato lines.

1. PCR system (total volume 20 μL)

10 μL Taq Master Mix, 1 μL genomic DNA template, 0.5 μL forward primer (10 μM), 0.5 μL reverse primer (10 μM), supplemented with 8 μL deionized water to a final volume of 20 μL.

2. Touchdown PCR thermal profile

Initial denaturation: 95 °C for 8 min

Cycle 1 (3 cycles):95 °C for 30 s; 65 °C for 30 s; 72 °C for 30 s

Cycle 2 (3 cycles):95 °C for 30 s; 63 °C for 30 s; 72 °C for 30 s

Cycle 3 (3 cycles):95 °C for 30 s; 61 °C for 30 s; 72 °C for 30 s

Cycle 4 (3 cycles):95 °C for 30 s; 59 °C for 30 s; 72 °C for 30 s

Cycle 5 (3 cycles):95 °C for 30 s; 57 °C for 30 s; 72 °C for 30 s

Cycle 6 (20 cycles):95 °C for 30 s; 55 °C for 30 s; 72 °C for 30 s

Final extension: 72 °C for 5 min

Hold: 25 °C for 5 min

3. Gene-specific primers for hLF amplification

hLF-F: 5′-ttcatttggagagaacacgggggac-3′

hLF-R: 5′-ctggagacataagagctttagc-3′

The predicted amplified fragment length was 577 bp.

4. Amplicon detection

PCR products were separated on a 1% agarose gel and visualized using a gel imaging system.

Supplemental Method S3. Total RNA extraction

Approximately 100 mg fresh tomato leaf tissue was ground into fine powder in liquid nitrogen. Total RNA was isolated via a modified CTAB method, followed by DNase I digestion to eliminate residual genomic DNA contamination. Briefly, the ground tissue powder was mixed with pre-warmed CTAB lysis buffer supplemented with β-mercaptoethanol and incubated at 65 °C. After phase separation with chloroform–isoamyl alcohol, RNA was precipitated with lithium chloride, washed with 70% ethanol, and dissolved in RNase-free water. All RNA samples were treated with DNase I at 37 °C to remove genomic DNA impurities, then the enzyme was inactivated. RNA purity and concentration were determined using a micro-spectrophotometer, and RNA integrity was checked by agarose gel electrophoresis for subsequent RT-qPCR analysis.

Supplemental Method S4. Screening for multi-allelic alleles and cell line database matching

1. Detailed STR genotyping results

HFL-1 human fetal lung fibroblast cells used in this study were subjected to full STR authentication by Jiangsu KeyGEN BioTECH Co., Ltd. A total of 21 human-specific STR loci plus the sex-determining Amelogenin (AMEL) locus were amplified and genotyped, including D5S818, D13S317, D7S820, D16S539, VWA, TH01, TPOX, CSF1PO, D12S391, FGA, D2S1338, D21S11, D18S51, D8S1179, D3S1358, D6S1043, PENTAE, D19S433, PENTAD, D1S1656 and AMEL.

For every detected locus, only one or two distinct allele peaks were observed; no loci displayed three or more allelic fragments (defined as multi-allelic alleles in cell authentication standards ANSI/ATCC ASN0002-2022). No cross-contamination with other cell lines or abnormal aneuploid allelic patterns was detected.

2. Procedures for multi-allelic allele screening

Genomic DNA was extracted from HFL-1 cell pellets, and multiplex STR amplification kits were used to co-amplify all target loci in a single PCR reaction.

Capillary electrophoresis was performed to separate amplified fragments, and raw fluorescence peak signals were analyzed by professional genotyping software.

Technicians manually reviewed the electrophoretogram of each locus: a locus was defined as multi-allelic if ≥3 independent allele peaks with valid signal intensity appeared simultaneously. All 22 loci were screened one by one for extra allelic peaks.

According to the ATCC standard, multi-allelic patterns indicate cell cross-contamination, mixed cell populations or genetic instability; none of these abnormal patterns were identified across all tested loci in our HFL-1 sample.

3. Cell line matching search workflow

The STR genotype data of our HFL-1 cells were imported into DSMZ official cell line matching tools, which integrate public STR datasets from ATCC, DSMZ, JCRB, RIKEN and ECACC, covering more than 2490 human cell lines.

The matching evaluation value (EV) was calculated to quantify the consistency between the sample STR profile and database reference profiles. An EV ≥ 80% indicates a high-probability identical cell line per ANSI/ATCC ASN0002-2022 criteria.

The query returned a single fully matched entry labeled HFL1 (catalog number CCL-153) in the DSMZ database, with an EV value of 1.0 (100% identity). The core shared loci with identical allelic profiles between our sample and the standard DSMZ HFL1 reference are listed as follows: D5S818 (12,12), D13S317 (11,12), D7S820 (9,10), D16S539 (9,11), VWA (17,17), TH01 (7,9),

AMEL (X,Y), TPOX (6,9), and CSF1PO (10,12). The remaining loci (D12S391, FGA, D2S1338, D21S11, D18S51, D8S1179, D3S1358, D6S1043, PENTAE, D19S433, PENTAD, D1S1656) are not included in the public DSMZ reference dataset for HFL1 and were thus excluded from matching scoring.
